# Growth-Associated Reorganization of Gill and Skin-Mucus Microbiota in Cultured *Decapterus maruadsi*: Contrasting Occurrence–Abundance Patterns

**DOI:** 10.3390/vetsci13101073

**Published:** 2026-10-10

**Authors:** Xiaoping Wu, Fangyu Yuan, Linkai Chen, Zhaokai Yu, Hancheng Hong, Ding Li, Weiqing Huang

**Affiliations:** 1 College of Marine Sciences, Ningde Normal University, Ningde 352100, China; wuxiaoping1216@163.com (X.W.); junbva1012@163.com (L.C.); kskbl0914@163.com (Z.Y.); thunder20050523@163.com (H.H.); 2 Engineering Research Center of Mindong Aquatic Product Deep-Processing, Ningde 352100, China; 3 School of Medicine, Fuzhou University, Fuzhou 350108, China; t25116@fzu.edu.cn

**Keywords:** *Decapterus maruadsi*, gill microbiota, skin mucus, growth stage, microbial succession, persistent ASVs

## Abstract

External mucosal microbiota form important interfaces between fish and the aquatic environment, yet their variation during host growth remains poorly understood. We characterized the gill and skin-mucus microbiota of cultured *Decapterus maruadsi* across three successive body-size stages (4.98–6.81 g, 16.67–19.63 g, and 27.30–28.66 g) using 16S rRNA gene amplicon sequencing. Fish were sampled from the same cohort maintained in the same culture system, and two supplied-seawater samples were included as environmental references. Factorial multivariate analysis indicated significant effects of mucosal tissue, sampling stage, and their interaction on community composition. Broad taxonomic structure remained dominated by *Pseudomonadota*, whereas extensive differentiation occurred at finer taxonomic resolutions. Skin-mucus richness decreased across successive sampling stages, whereas no corresponding alpha-diversity difference was detected in the gill. Host-wide prevalence analysis revealed contrasting changes in the abundance contribution of recurrent ASVs between the two mucosal compartments; however, tissue-specific sensitivity analyses showed that occurrence categories depended partly on the sample set used to define prevalence. The same dominant *Limimaricola* ASV occurred at high abundance in one supplied-seawater sample and in middle-stage skin mucus, indicating environmental microbial input as a plausible contributor to the observed composition. Overall, this study provides a descriptive baseline for stage-associated variation in the external mucosal microbiota of one cultured *D. maruadsi* cohort and highlights distinct responses of gill and skin-mucus communities without attributing these changes solely to host growth.

## Simple Summary

Fish gills and skin mucus are continuously exposed to surrounding water, but they provide different biological surfaces for microbial colonization. We examined the gill and skin-mucus microbiota of cultured *Decapterus maruadsi* at three successive growth stages from the same cohort. The two mucosal habitats changed differently during growth. In the gill, a small group of microorganisms that occurred widely across the complete dataset contributed an increasingly large fraction of community abundance from the early to middle stage and remained important at the late stage. Skin mucus followed a different, non-linear pattern: the middle-stage community was strongly dominated by microorganisms with intermediate occurrence frequencies, before high-prevalence microorganisms again became abundant at the late stage. Broad bacterial groups remained similar among samples, but extensive differences emerged at the genus and amplicon sequence variant (ASV) levels. These findings show that gill and skin-mucus microbiota undergo distinct patterns of growth-associated reorganization rather than a uniform developmental process.

## 1. Introduction

Fish-associated microbiota are integral components of aquatic host–microbe systems and are shaped jointly by host biology and environmental exposure [1,2]. Unlike terrestrial vertebrates, fish remain immersed in microbe-rich water throughout life, creating continuous opportunities for microbial immigration to external mucosal surfaces. Nevertheless, host-associated microbial communities are not simple reflections of surrounding water. Anatomical niche, epithelial characteristics, mucus composition, nutrient availability, immune regulation, physicochemical conditions, and microbial interactions can all contribute to differentiation among host-associated assemblages [1,2,3].

The gill and skin mucus represent two major external mucosal habitats but differ substantially in physiological function and local environmental conditions. The gill is a highly vascularized organ involved in respiratory gas exchange, osmoregulation, ion transport, acid–base regulation, and nitrogenous waste excretion [4,5]. These functions create a specialized interface that can support microbial communities distinct from those in surrounding water [4,6,7]. Skin mucus, by contrast, forms a continuously renewed external barrier at the body surface and provides a dynamic microbial habitat influenced by both host-derived secretions and environmental contact [3,8,9,10]. Accordingly, gill and skin mucus can differ markedly in microbial composition even within the same host species or culture environment [6,7,8,11,12,13,14].

External mucosal microbiota may also vary during host growth and development. Body-size increase is accompanied by longer cultivation duration and changes in feeding intensity, metabolism, immune development, epithelial properties, mucus production, and cumulative environmental exposure. Temporal, environmental, and biological factors have been shown to influence the mucosal microbiota of wild Pacific chub mackerel (*Scomber japonicus*) [15]. Age-associated changes in skin and gill microbial communities have also been reported in farmed seabass and seabream [16]. In yellowtail kingfish (*Seriola lalandi*), water, feed, and the hatchery environment contributed to mucosal microbiome colonization across ontogeny [17]. Temporal restructuring of skin and gill microbiota has likewise been documented in Atlantic salmon (*Salmo salar*) maintained in a recirculating aquaculture system [18]. Together, these studies indicate that external mucosal microbial communities can remain dynamic throughout host development.

Most developmental microbiome studies emphasize alpha diversity, beta diversity, and taxonomic composition. These analyses are essential for describing community structure but provide limited information about how consistently individual microbial members occur across a sample set. Prevalence-based classification provides an additional ecological dimension by distinguishing low-, intermediate-, and high-occurrence taxa. In this framework, transient ASVs are detected in a small fraction of samples, intermittent ASVs occur at intermediate prevalence, and persistent ASVs recur across most samples [19]. These terms describe occurrence across the analyzed dataset and do not establish longitudinal residence within an individual fish.

This distinction is particularly relevant for external mucosal habitats. Continuous environmental exposure can generate a large pool of low-prevalence microorganisms, while a much smaller set of recurrent ASVs may account for a disproportionate share of total community abundance. Consequently, numerical representation and abundance dominance can become strongly decoupled. Integrating taxonomic composition, fine-scale ASV turnover, and prevalence-based occurrence structure can therefore reveal community changes that are not apparent from diversity metrics alone.

Taxonomic resolution is also important. Broad bacterial groups may remain relatively conserved while extensive reorganization occurs among genera and individual ASVs. Comparative studies across marine fishes have shown that host biology, anatomical niche, habitat, and environmental conditions can generate marked fine-scale differentiation despite similarity at higher taxonomic ranks [6,7,8,11,13]. Thus, a broadly conserved phylum-level framework does not necessarily indicate equivalent microbial community organization.

*Decapterus maruadsi* is a carangid fish widely distributed in the western Pacific and represents an important fishery resource in China [20]. External mucosal microbiome succession across growth stages of *D. maruadsi* remains poorly characterized. Gill and skin-mucus microbiota were analyzed using 16S rRNA gene amplicon sequencing, while truck-delivered seawater from the same provider used throughout cultivation was included as an environmental reference. We evaluated community structure, multilevel taxonomic composition, shared and group-specific membership, dominant ASV distributions, candidate group-associated taxa, and dataset-wide occurrence classes. The central question was whether the gill and skin-mucus microbiota follow similar or distinct patterns of microbial reorganization during host growth.

## 2. Materials and Methods

### 2.1. Experimental Design and Sample Collection

Cultured *D. maruadsi* used in this study originated from the same cohort and were continuously maintained in the same tank-based culture system under continuous aeration throughout the study period. All fish received the same formulated diet throughout the study period and were fed twice daily. Feed formulation remained unchanged during cultivation, whereas feeding quantity was adjusted according to fish growth and feeding requirements. A fixed feeding rate expressed as a percentage of body weight was not systematically recorded.

The same cohort was sampled successively at approximately 3, 4, and 5 months of age. The early stage (4.98–6.81 g) was sampled on 11 November 2025, the middle stage (16.67–19.63 g) on 17 December 2025, and the late stage (27.30–28.66 g) on 25 January 2026. The early-to-middle and middle-to-late sampling intervals were therefore 36 and 39 days, respectively. These groups represented successive growth-associated sampling stages of the same continuously reared cohort rather than independently reared experimental populations. Concurrent measurements of water temperature, salinity, dissolved oxygen, pH, ammonia, and nitrite were not systematically available for the three sampling periods.

At each sampling stage, 15 fish showing no obvious external lesions, abnormal behavior, or visible signs of disease were collected and divided into three subsets of five fish. Gill samples from the early, middle, and late stages were designated Gill-Early (BLEGS), Gill-Mid (BLMGS), and Gill-Late (BLLGS), respectively, whereas the corresponding skin-mucus samples were designated Mucus-Early (BLEGTB), Mucus-Mid (BLMGTB), and Mucus-Late (BLLGTB).

Fish were euthanized by immersion in tricaine methanesulfonate (MS-222; 200 mg L^−1^) before tissue collection. Skin mucus was collected aseptically by swabbing the abdominal body surface with sterile cotton swabs while avoiding contact with the anus and gill region. Gill tissue was subsequently collected without restriction to a specific gill arch using sterile scissors and forceps and was gently rinsed with sterile water before pooling. For each subset, gill tissues obtained from five fish were pooled before DNA extraction, and skin-mucus material obtained from the same five fish was pooled separately. Thus, each gill sample had a corresponding skin-mucus sample derived from the same subset of fish. Three pooled gill samples and three paired pooled skin-mucus samples were obtained at each sampling stage. The pooled sample, rather than the individual fish, was treated as the analytical unit.

### 2.2. Supplied Seawater Sampling and Genomic DNA Extraction

Seawater used in the culture system was delivered to the facility by tanker truck. Two supplied-seawater samples, BLY_ST1 and BLY_ST2, were obtained from two independent tanker-delivery events during the cultivation period. Because seawater replacement occurred repeatedly during routine culture management, the exact sampling dates of these two reference samples could not be retrospectively resolved. Consequently, BLY_ST1 and BLY_ST2 could not be assigned to specific fish sampling stages and were treated only as environmental reference samples rather than as stage-matched water samples.

Approximately 2 L of each seawater sample was filtered through a sterile 0.22 μm membrane to concentrate microbial biomass. The 0.22 μm pore size was selected to maximize retention of bacterial cells from seawater, and microbial biomass retained on the membrane, rather than the filtrate, was subsequently used for DNA extraction. Microbial genomic DNA was extracted using the DNeasy PowerSoil Pro Kit (QIAGEN GmbH, Hilden, Germany) according to the manufacturer’s protocol. Gill tissues were aseptically homogenized before DNA extraction. DNA from skin mucus was extracted from material retained on the sterile cotton swabs, whereas DNA from supplied seawater was extracted from microbial biomass retained on the filtration membranes.

The concentration of extracted DNA was determined using a Qubit 3.0 Fluorometer (Life Technologies, Thermo Fisher Scientific, Waltham, MA, USA) and the Equalbit 1× dsDNA HS Assay Kit (Vazyme, Nanjing, China). DNA integrity was assessed using 1.0% agarose gel electrophoresis, whereas DNA purity was evaluated using a NanoDrop ND-2000 spectrophotometer (Thermo Scientific, Waltham, MA, USA). These quality-control assessments were performed before subsequent PCR amplification.

### 2.3. 16S rRNA Gene Amplification and Illumina Sequencing

The V4 region of the prokaryotic 16S rRNA gene was amplified using the primer pair 515FmodF (5′-GTGYCAGCMGCCGCGGTAA-3′) and 806R (5′-GGACTACHVGGGTWTCTAAT-3′). PCR reactions were performed in a total volume of 20 μL containing 10 ng of template DNA, 0.8 μL of each primer (10 μM), 4 μL of 5× FastPfu buffer, 0.4 μL of FastPfu polymerase, 0.2 μL of bovine serum albumin, and 250 μM dNTPs. PCR cycling consisted of an initial denaturation at 95 °C for 3 min, followed by 29 cycles of denaturation at 95 °C for 30 s, annealing at 55 °C for 30 s, and extension at 72 °C for 45 s, with a final extension at 72 °C for 10 min. PCR products were examined by agarose gel electrophoresis, purified, quantified, and pooled in equimolar proportions. Purified amplicons were sequenced using an Illumina MiSeq PE300 platform according to the standard protocols of Majorbio Bio-Pharm Technology Co., Ltd. (Shanghai, China). DNA extraction blanks and no-template PCR negative controls were included during DNA extraction and amplification, respectively, to monitor potential laboratory contamination. These negative controls were not included in the final sequencing library. Consequently, sequence-based retrospective identification and removal of potential reagent-derived contaminants could not be performed. Because the present amplicon-sequencing approach specifically targeted the prokaryotic 16S rRNA gene, viral communities were not assessed and cannot be evaluated using the current dataset.

### 2.4. Sequence Processing and Taxonomic Assignment

Raw sequencing reads were processed using the QIIME 2 (version 2020.2) pipeline [21]. Primer sequences were removed using Cutadapt (version 2.8) [22]. Quality filtering, denoising, paired-end merging, and chimera removal were performed using DADA2 (version 2020.2.0) [23] to generate amplicon sequence variants (ASVs). Representative ASV sequences were aligned using MAFFT (version 7.310) [24], and phylogenetic reconstruction was performed using FastTree2 (version 2.1.10) [25]. Taxonomic assignment was performed against the SILVA Release 138 reference database [26]. ASVs assigned to chloroplasts, mitochondria, eukaryotes, or other non-target lineages were removed before downstream analyses (Supplementary Table S1). The resulting filtered ASV abundance table was used for rarefaction, beta-diversity, taxonomic composition, shared-taxon, heatmap, differential-abundance, and occurrence-frequency analyses.

### 2.5. Diversity and Community-Structure Analyses

Rarefaction curves based on Chao richness [27], Shannon diversity [28], and Good’s coverage were generated to evaluate sequencing completeness. These curves were used to assess sampling sufficiency rather than to test formal group-level alpha-diversity differences. Beta diversity was assessed using Bray–Curtis dissimilarity calculated from ASV relative abundance profiles [29]. Principal coordinate analysis (PCoA) was used to visualize compositional relationships among BLEGS, BLMGS, BLLGS, BLEGTB, BLMGTB, and BLLGTB. Overall differences among the six host-associated groups were evaluated using analysis of similarities (ANOSIM) with 999 permutations [30]. Statistical significance was defined as *p* < 0.05. In addition to the overall six-group ANOSIM, a factorial PERMANOVA based on Bray–Curtis dissimilarity was performed to evaluate the effects of mucosal tissue, sampling stage, and the tissue × stage interaction. Marginal sums of squares were used. Because gill and skin-mucus samples within each pooled subset originated from the same five fish, permutations were restricted to preserve the paired sampling structure.

### 2.6. Taxonomic Composition, Shared Taxa, and Heatmap Visualization

Microbial community composition was summarized at the phylum, class, order, family, and genus levels using relative abundance. Shared and group-specific taxa were evaluated using Venn diagrams at the phylum, family, genus, and ASV levels. A taxon was considered present in a group when it was detected in at least one sample assigned to that group. For heatmap visualization, the 50 most abundant ASVs were selected, and their abundance values were log-transformed before hierarchical visualization. The heatmap was therefore used to compare relative abundance patterns among samples and groups rather than to derive absolute abundance values directly from the color scale.

### 2.7. Occurrence-Frequency Classification of ASVs

Classification was performed at the individual-ASV level using sample-level presence–absence information; sequencing reads were not pooled across samples before prevalence calculation. After classification, relative abundance contributions were calculated separately for each pooled sample and subsequently summarized within each sampling group. The primary occurrence-frequency analysis was restricted to the 18 host-associated samples, consisting of nine gill and nine skin-mucus samples. The two supplied-seawater samples were excluded from prevalence classification because they served only as environmental references and were not temporally matched to the fish sampling stages. For each ASV, prevalence was calculated as the proportion of the 18 host-associated samples in which that ASV was detected. ASVs were operationally classified as transient (prevalence < 0.2), intermittent (0.2 ≤ prevalence ≤ 0.8), or persistent (prevalence > 0.8). Under this definition, persistent ASVs were detected in at least 15 of the 18 host-associated samples [19]. These cut-offs were used as analytical boundaries to distinguish low-, intermediate-, and high-prevalence ASVs within the present dataset and were not intended to represent universal biological definitions of transient, intermittent, or core microbiome membership. Because prevalence classification can depend on the selected thresholds, a sensitivity analysis was additionally performed using alternative cut-offs of <0.25, 0.25–0.75, and >0.75.

Because prevalence categories can depend on the sample set used to define occurrence, complementary sensitivity analyses were performed by recalculating prevalence separately within the nine gill samples and the nine skin-mucus samples. Under these tissue-specific analyses, persistent ASVs were those detected in at least eight of nine samples. In addition, the combined 18-host analysis was repeated using alternative prevalence thresholds of <0.25, 0.25–0.75, and >0.75. These sensitivity analyses were used to evaluate the dependence of occurrence–abundance patterns on analytical scope and threshold choice. The terms transient, intermittent, and persistent are operational prevalence categories and do not imply longitudinal residence of individual microbial populations within the same fish.

### 2.8. Statistical Analysis

Differences in the relative abundance of genera and ASVs among the six host-associated groups were evaluated using the Kruskal–Wallis H test. To account for multiple comparisons, Benjamini–Hochberg false-discovery-rate correction was applied separately across the complete set of genus-level tests and ASV-level tests. Both nominal *p* values and FDR-adjusted q values are reported. Taxa with nominal *p* < 0.05 but adjusted q ≥ 0.05 were interpreted as exploratory candidates rather than statistically confirmed differential taxa. Given the limited number of pooled samples per group (n = 3), taxon-level results were interpreted cautiously.

## 3. Results

### 3.1. Sequencing Coverage and Overall Community Differentiation

Across the 20 analyzed samples, 1,831,181 optimized sequences were obtained, ranging from 43,410 to 151,389 sequences per sample. After denoising, 1,558,959 sequences remained, with individual sample depths ranging from 38,746 to 110,538 sequences. All samples were subsequently standardized to a final depth of 35,884 sequences for diversity analysis. Rarefaction curves based on Chao richness, Shannon diversity, and Good’s coverage approached stable plateaus with increasing sequencing depth (Figure S1). Chao and Shannon profiles varied among individual samples, whereas Good’s coverage approached 1.0 across the dataset. These patterns indicated that sequencing depth was sufficient to capture the major detectable microbial diversity in the analyzed samples. Alpha-diversity patterns differed between the two mucosal compartments. No statistically significant stage-level differences were detected in the gill for Chao1 richness (exact Kruskal–Wallis *p* = 0.721), Shannon diversity (*p* = 0.629), or observed ASV richness (*p* = 0.721). In contrast, skin-mucus richness decreased across successive sampling stages. Chao1 richness differed among the three mucus stages (exact Kruskal–Wallis *p* = 0.0286), as did observed ASV richness (*p* = 0.0107), whereas Shannon diversity did not differ significantly (*p* = 0.132). Sampling stage was also negatively correlated with Chao1 richness (Spearman ρ = −0.843, *p* = 0.0043) and observed ASV richness (ρ = −0.896, *p* = 0.0011) in skin mucus.

**Figure 1:**
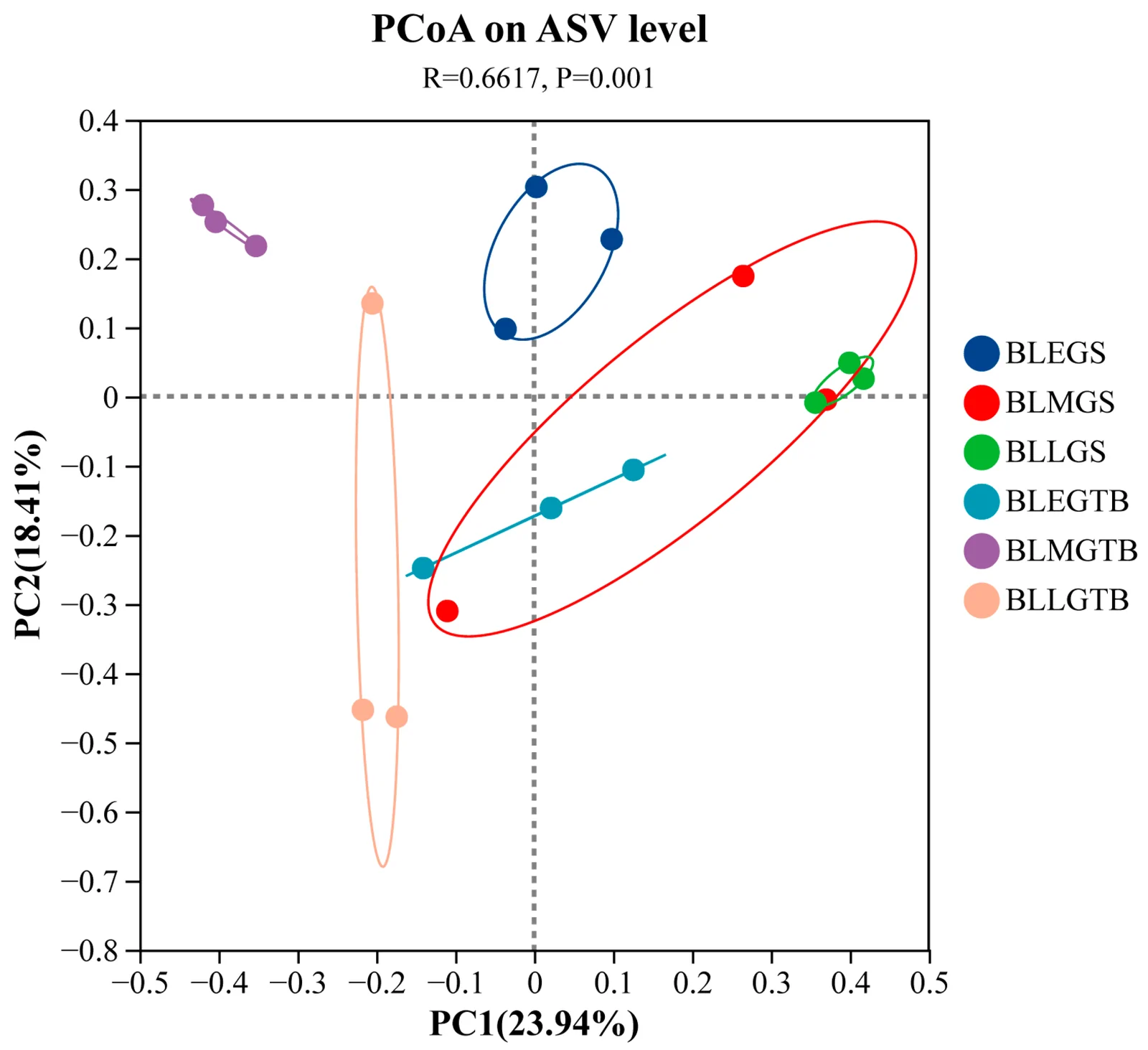
Principal coordinate analysis (PCoA) of ASV-level microbial community structure across six gill and skin-mucus groups of *D. maruadsi*. Bray–Curtis dissimilarity was calculated from ASV relative abundance profiles. BLEGS, BLMGS, and BLLGS represent gill samples from fish weighing 4.98–6.81 g, 16.67–19.63 g, and 27.30–28.66 g, respectively; BLEGTB, BLMGTB, and BLLGTB represent the corresponding skin-mucus groups.

ASV-level PCoA revealed pronounced compositional differentiation among the six host-associated groups (Figure 1). PC1 explained 23.94% of total community variation and PC2 explained 18.41%, together accounting for 42.35% of the variation represented by the first two axes. ANOSIM confirmed significant overall differentiation among groups (R = 0.6617, *p* = 0.001). BLMGS samples were visibly more dispersed in ordination space than several of the other groups, indicating considerable within-group heterogeneity at the middle gill stage. Given the small number of pooled samples per group, the ordination is interpreted primarily as a visualization of compositional relationships rather than evidence of uniformly tight group clustering.

The three gill groups occupied partially distinct regions of ordination space. BLEGS samples were positioned mainly near the upper-central region, BLMGS occupied a broader central-to-right region, and BLLGS was concentrated toward positive PC1 values. Skin-mucus samples showed a different configuration: BLEGTB occupied mainly negative PC2 values, BLMGTB formed a compact cluster in the upper-left region, and BLLGTB shifted strongly toward negative PC2. Thus, the ordination patterns differed between mucosal habitats, with the strongest skin-mucus displacement occurring at the middle and late stages. Factorial PERMANOVA identified significant effects of mucosal tissue (pseudo-F = 3.110, *p* = 0.0156), sampling stage (pseudo-F = 1.934, *p* = 0.0036), and the tissue × stage interaction (pseudo-F = 2.988, *p* = 0.0036) on Bray–Curtis community composition. The significant interaction indicates that changes across sampling stages differed between the gill and skin-mucus compartments.

### 3.2. Broad Taxonomic Conservation Coexisted with Marked Sample-Level Variation

**Figure 2:**
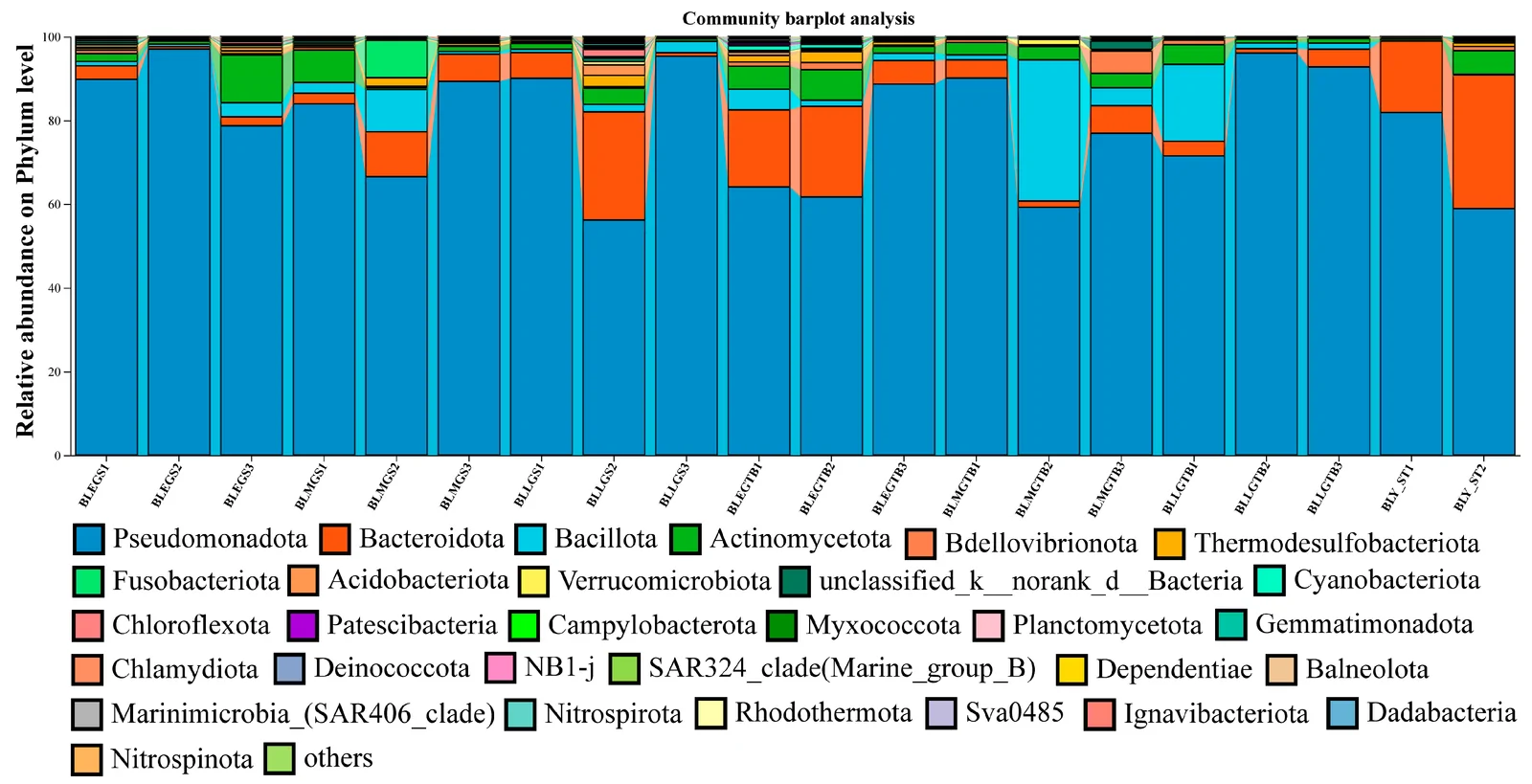
Phylum-level microbial community composition across gill, skin-mucus, and supplied-seawater samples of *D. maruadsi*. Stacked bars show relative abundances of the dominant bacterial phyla in each sample.

*Pseudomonadota* represented the dominant phylum in nearly all gill, skin-mucus, and supplied-seawater samples (Figure 2). However, the magnitude of *Pseudomonadota* dominance varied substantially. BLEGS2 and several late-stage skin-mucus samples were strongly dominated by *Pseudomonadota*, whereas BLLGS2, BLMGTB2, and BLY_ST2 contained larger contributions from secondary phyla. *Bacteroidota* was particularly prominent in BLLGS2, BLEGTB1–2, and BLY_ST2, while *Bacillota* increased markedly in BLMGTB2. *Actinomycetota* and several lower-abundance phyla contributed variably among individual samples. Thus, growth was not accompanied by wholesale replacement of dominant phyla; instead, a broadly *Pseudomonadota*-dominated framework persisted while the contribution of secondary phyla changed among stages and mucosal sites.

At the class level (Figure S2), *Alphaproteobacteria* and *Gammaproteobacteria* constituted the major components of most communities, but their balance varied strongly among samples. BLEGS2 was strongly dominated by *Gammaproteobacteria*, whereas BLEGS1 and BLEGS3 contained larger *Alphaproteobacteria* fractions. BLMGTB1 was predominantly *Alphaproteobacteria*, while BLMGTB2 contained a conspicuous *Bacilli* component. BLLGTB2 and BLLGTB3 were dominated by *Gammaproteobacteria*. The supplied-seawater samples also differed from each other, with BLY_ST1 showing strong *Alphaproteobacteria* dominance and BLY_ST2 containing a more mixed class-level profile.

Order- and family-level profiles provided additional resolution (Figures S3 and S4). *Rhodobacterales*, *Enterobacterales*, *Hyphomicrobiales*, *Burkholderiales*, *Pseudomonadales*, and *Flavobacteriales* were major orders, but their relative contributions differed markedly among stages. At the family level, *Paracoccaceae*, *Vibrionaceae*, *Beijerinckiaceae*, *Flavobacteriaceae*, *Moraxellaceae*, *Pseudomonadaceae*, *Exiguobacteraceae*, *Comamonadaceae*, *Burkholderiaceae*, and several additional families contributed substantially. BLMGTB1 and BLMGTB3 contained large *Paracoccaceae* fractions, whereas BLMGTB2 combined *Paracoccaceae* with a marked *Exiguobacteraceae* component. In contrast, BLLGTB2 and BLLGTB3 displayed strong *Vibrionaceae* dominance. These patterns show that increasingly pronounced differentiation emerged below the phylum level.

### 3.3. Genus-Level Composition Revealed Distinct Gill and Skin-Mucus Reorganization

**Figure 3:**
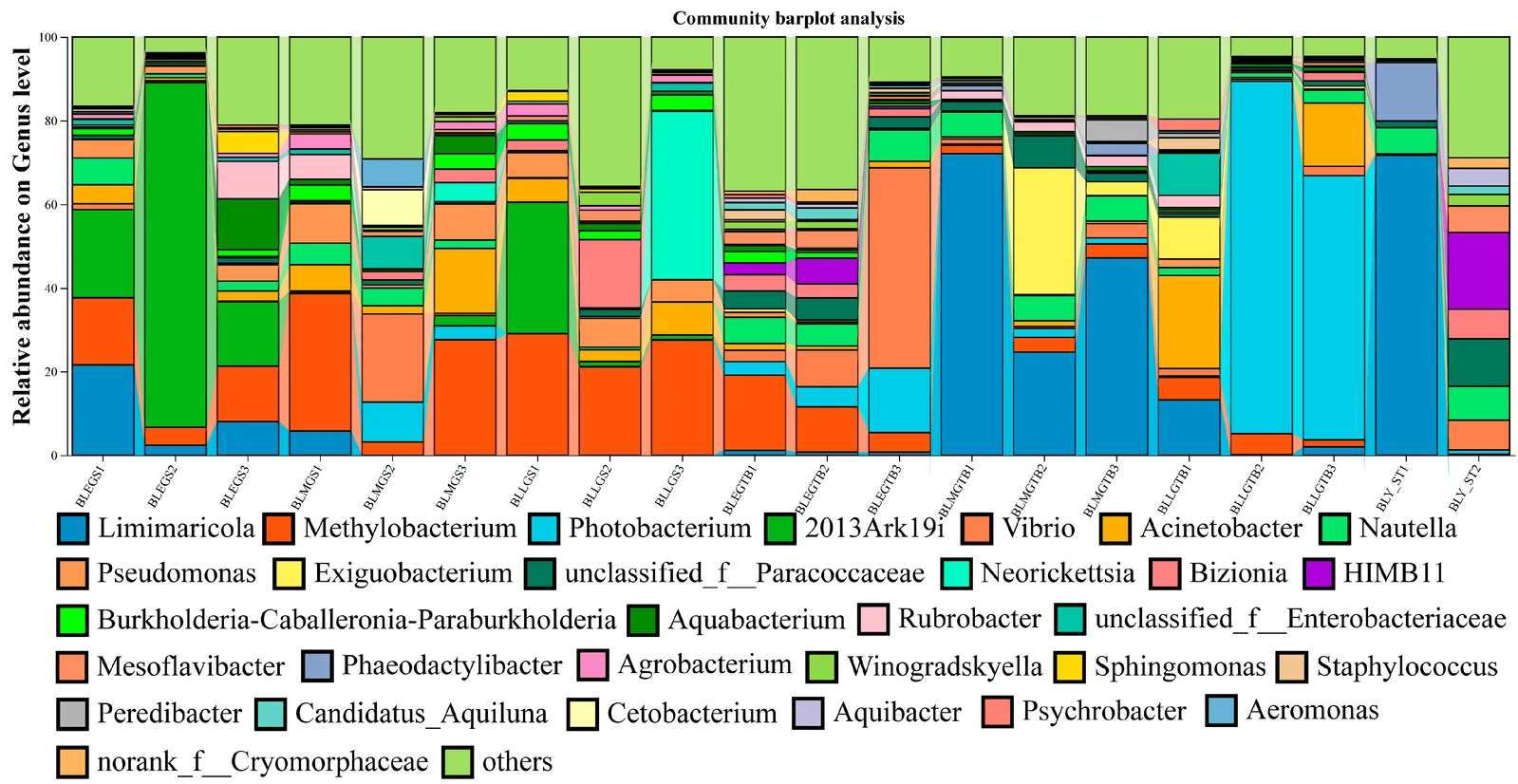
Genus-level microbial community composition across gill, skin-mucus, and supplied-seawater samples of *D. maruadsi*. Stacked bars show relative abundances of the dominant genera or genus-level lineages in each sample.

Genus-level composition showed substantially stronger differences than were apparent at the phylum level (Figure 3). Major detected genera or genus-level lineages included *Limimaricola*, *Methylobacterium*, *Photobacterium*, 2013Ark19i, *Vibrio*, *Acinetobacter*, *Nautella*, *Pseudomonas*, *Exiguobacterium*, *Neorickettsia*, HIMB11, *Burkholderia-Caballeronia-Paraburkholderia*, *Aquabacterium*, *Rubrobacter*, *Mesoflavibacter*, *Phaeodactylibacter*, *Agrobacterium*, *Winogradskyella*, *Sphingomonas*, *Staphylococcus*, *Peredibacter*, *Candidatus Aquiluna*, *Cetobacterium*, *Aquibacter*, *Psychrobacter*, and *Aeromonas*. The labels 2013Ark19i and HIMB11 represent provisional genus-level lineage designations retained from the SILVA Release 138 taxonomy rather than formally described genus names.

The early-stage gill microbiota was heterogeneous among pooled samples. BLEGS2 was strongly dominated by 2013Ark19i, whereas BLEGS1 and BLEGS3 contained more mixed assemblages with contributions from *Limimaricola*, *Methylobacterium*, 2013Ark19i, *Vibrio*, *Nautella*, and other genera. Thus, 2013Ark19i was characteristic of the BLEGS group at the group level but was not uniformly dominant across all three pools.

BLMGS displayed a different genus-level configuration. *Methylobacterium* contributed substantially to all three pools, while additional genera varied among samples. BLMGS1 contained a mixture of *Methylobacterium*, 2013Ark19i, *Vibrio*, *Nautella*, and other taxa; BLMGS2 included *Methylobacterium*, *Photobacterium*, *Acinetobacter*, and HIMB11; and BLMGS3 contained *Methylobacterium* together with *Acinetobacter*, *Pseudomonas*, and additional taxa. BLLGS represented another redistribution rather than a simple continuation of BLMGS. *Methylobacterium* remained prominent in BLLGS1 and BLLGS3, while BLLGS1 also retained a substantial 2013Ark19i contribution and BLLGS2 showed a broader mixed assemblage.

Skin mucus followed a different sequence. BLEGTB samples contained relatively mixed communities without a single genus dominating all pools. BLMGTB showed a conspicuous increase in *Limimaricola*: BLMGTB1 was strongly dominated by this genus, BLMGTB3 also contained a large *Limimaricola* fraction, and BLMGTB2 combined *Limimaricola* with a pronounced *Exiguobacterium* component. BLLGTB differed again. BLLGTB1 contained a mixed assemblage including *Limimaricola* and *Acinetobacter*, whereas BLLGTB2 and BLLGTB3 were strongly enriched in *Photobacterium*. The transition from BLMGTB to BLLGTB therefore involved substantial redistribution of dominant genera rather than persistence of the middle-stage taxonomic state.

The two supplied-seawater samples also differed strongly. BLY_ST1 was dominated by *Limimaricola*, whereas BLY_ST2 contained a more heterogeneous assemblage. Their divergence reinforces the use of BLST as an environmental reference rather than a stage-matched temporal water series.

### 3.4. Universal Overlap Declined Strongly with Increasing Taxonomic Resolution

**Figure 4:**
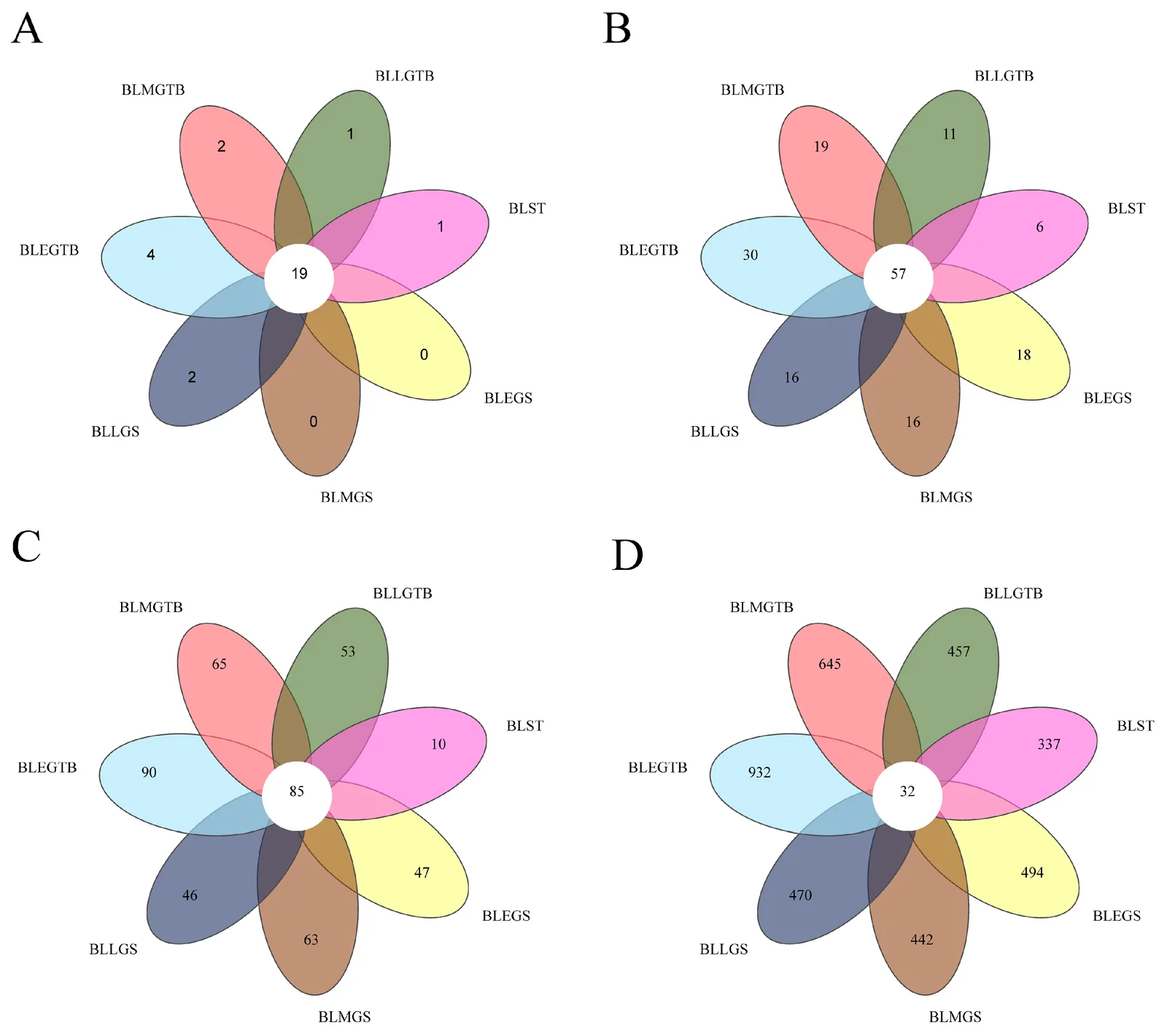
Shared and group-specific microbial taxa across gill, skin-mucus, and supplied-seawater groups of *D. maruadsi*. Venn diagrams show shared and group-specific taxa at the (**A**) phylum, (**B**) family, (**C**) genus, and (**D**) ASV levels. A taxon was considered present in a group when it was detected in at least one sample assigned to that group.

Venn analysis showed that the apparent degree of community overlap declined markedly with increasing taxonomic resolution (Figure 4). Nineteen phyla were shared across all seven groups, whereas group-specific phyla were uncommon: BLEGS and BLMGS contained no unique phyla, while BLLGS, BLEGTB, BLMGTB, BLLGTB, and BLST contained 2, 4, 2, 1, and 1 unique phyla, respectively.

At the family level, 57 families were shared among all groups, but group-specific membership increased. BLEGS, BLMGS, and BLLGS contained 18, 16, and 16 unique families, respectively; BLEGTB, BLMGTB, and BLLGTB contained 30, 19, and 11; and BLST contained six unique families. At the genus level, 85 genera were universally shared, whereas BLEGS, BLMGS, and BLLGS contained 47, 63, and 46 unique genera, and BLEGTB, BLMGTB, and BLLGTB contained 90, 65, and 53. BLST contained 10 unique genera.

Fine-scale differentiation was most pronounced at the ASV level. Only 32 ASVs were shared across all seven groups, while every group contained hundreds of group-specific ASVs. BLEGS, BLMGS, and BLLGS contained 494, 442, and 470 unique ASVs, respectively. The corresponding values for BLEGTB, BLMGTB, and BLLGTB were 932, 645, and 457, whereas BLST contained 337 unique ASVs. BLEGTB therefore contained the largest group-specific ASV pool among the host-associated communities. Collectively, these results demonstrate that broad taxonomic conservation coexisted with extensive fine-scale turnover. To further evaluate stage-associated ASV turnover within each mucosal compartment, shared-ASV patterns were analyzed separately for gill and skin mucus (Table S2). The gill contained 178 ASVs shared across all three sampling stages, while 730, 615, and 587 ASVs were specific to the early, middle, and late stages, respectively. Skin mucus contained 265 ASVs shared across all three stages, whereas the corresponding stage-specific ASV numbers decreased from 1223 in the early stage to 839 in the middle stage and 585 in the late stage. Pairwise shared-ASV proportions between adjacent stages are additionally provided in Table S2. These results indicate that stage-associated membership turnover occurred in both mucosal compartments, with a more pronounced decline in stage-specific ASV richness across successive skin-mucus stages (Supplementary Table S2).

### 3.5. Dominant ASVs and Candidate Group-Associated Taxa Revealed Fine-Scale Differentiation

**Figure 5:**
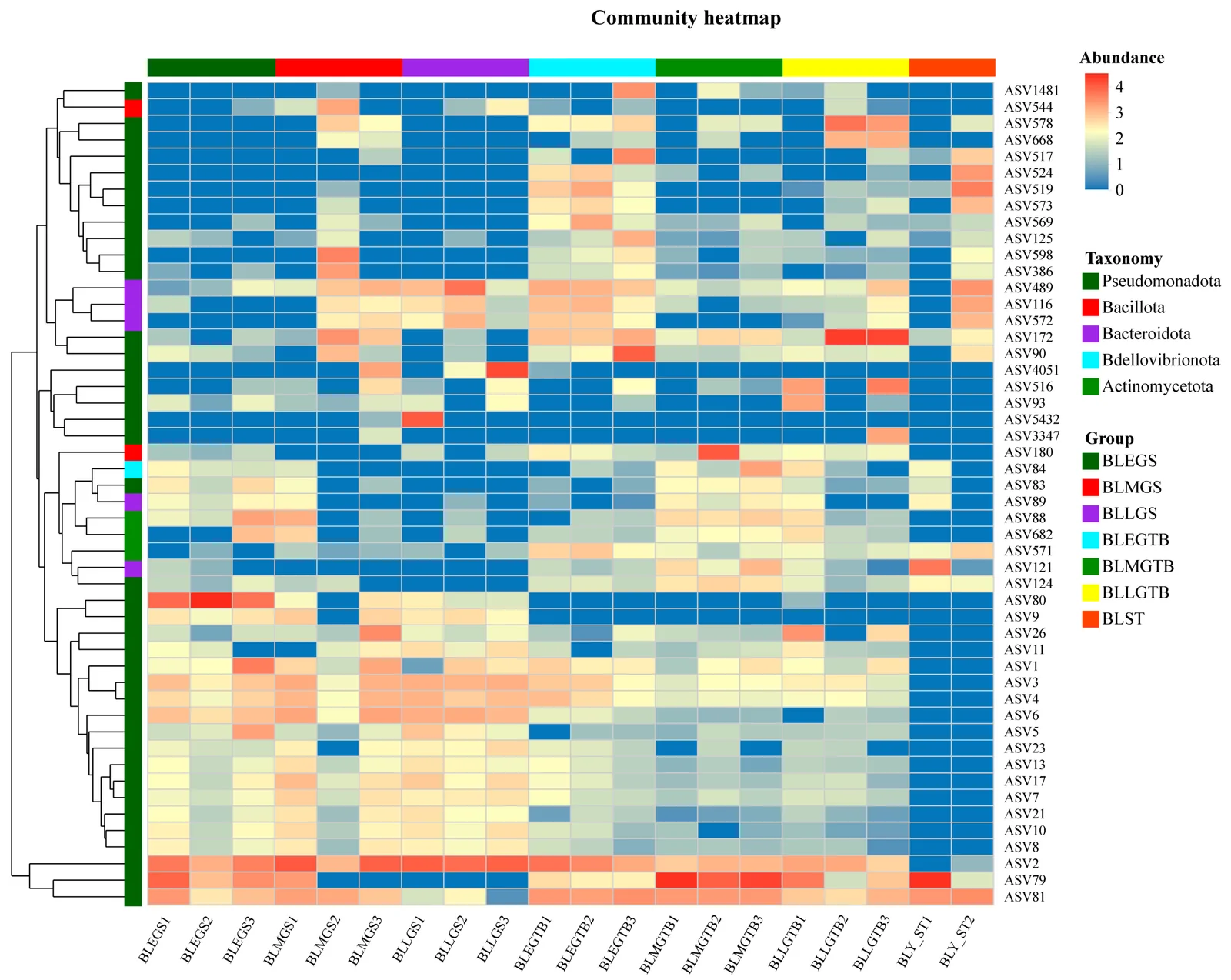
Heatmap of the 50 most abundant ASVs across gill, skin-mucus, and supplied-seawater samples of *D. maruadsi*. Columns represent individual samples and rows represent ASVs. Abundance values were log-transformed for visualization. The side annotation indicates ASV taxonomy at the phylum level, and the top annotation indicates sample group.

The Top-50 ASV heatmap further illustrated fine-scale variation among samples (Figure 5). Most high-abundance ASVs were assigned to *Pseudomonadota*, with additional representatives of *Bacillota*, *Bacteroidota*, *Bdellovibrionota*, and *Actinomycetota*. Several ASVs were broadly distributed among host-associated samples, whereas others showed strongly localized abundance patterns. ASV80 was particularly abundant in BLEGS, ASV79 and ASV180 showed pronounced representation in BLMGTB, and ASV172 was strongly represented in BLLGTB. Several water-associated ASVs also showed contrasting abundance between BLY_ST1 and BLY_ST2, consistent with the heterogeneity of the supplied-seawater reference.

**Figure 6:**
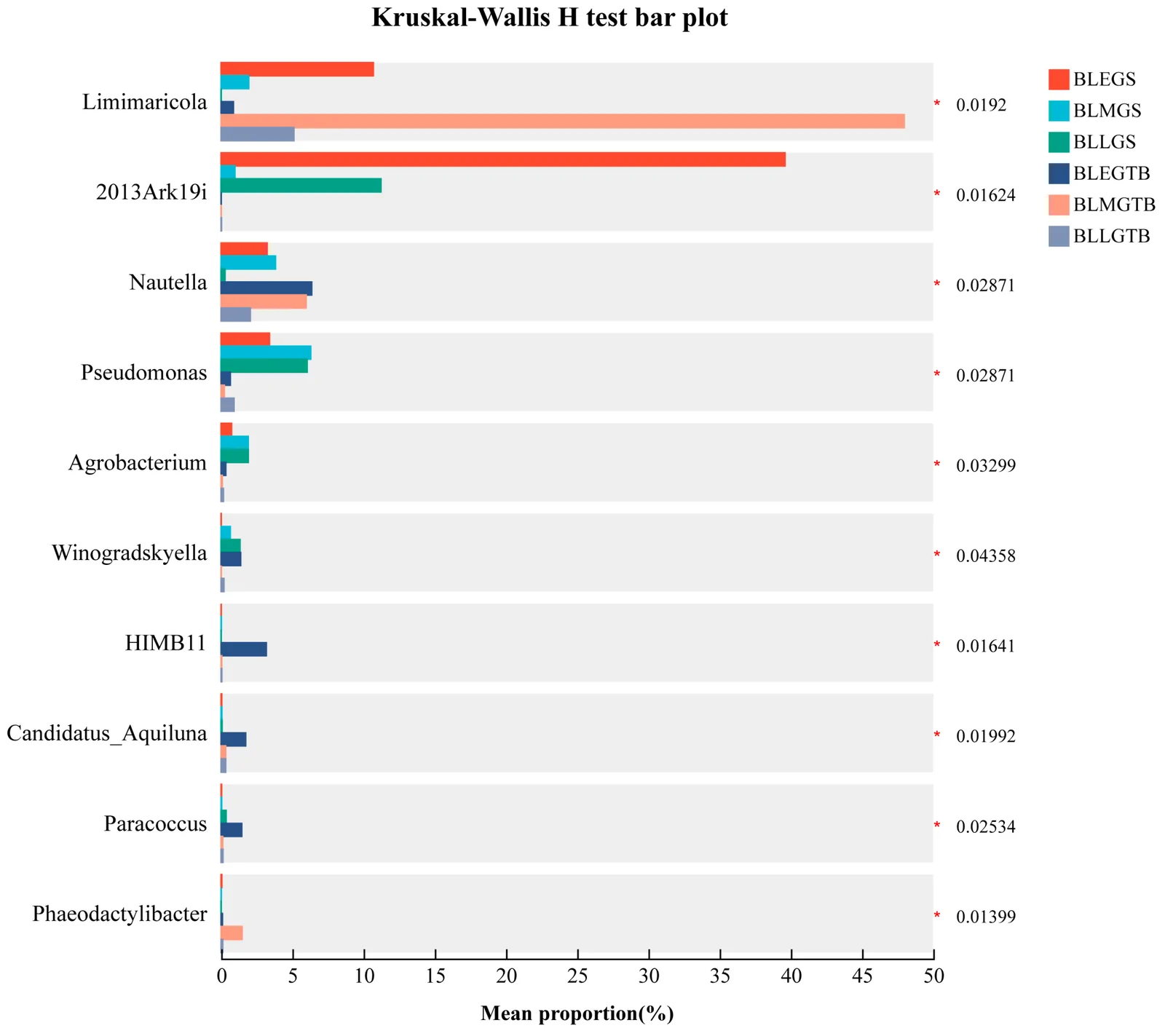
Candidate genera showing nominal group-level differences among gill and skin-mucus groups of *D. maruadsi*. Bars show mean relative abundance in BLEGS, BLMGS, BLLGS, BLEGTB, BLMGTB, and BLLGTB. Overall group differences were evaluated using the Kruskal–Wallis H test. Displayed *p* values are nominal and should be interpreted as exploratory. Corresponding group standard deviations and adjusted q values are provided in Table S3, whereas individual pooled-sample abundances are provided in Table S4. * *p* < 0.05.

Kruskal–Wallis analysis identified 10 genera with nominal group-level *p* values below 0.05 among the six host-associated groups (Figure 6). However, these taxa did not remain significant after Benjamini–Hochberg FDR correction and are therefore reported as exploratory group-associated features rather than statistically confirmed differential taxa. *Phaeodactylibacter* (*p* = 0.01399), *Paracoccus* (*p* = 0.02534), *Candidatus Aquiluna* (*p* = 0.01992), HIMB11 (*p* = 0.01641), *Winogradskyella* (*p* = 0.04358), *Agrobacterium* (*p* = 0.03299), *Pseudomonas* (*p* = 0.02871), *Nautella* (*p* = 0.02871), 2013Ark19i (*p* = 0.01624), and *Limimaricola* (*p* = 0.01920). *Limimaricola* showed its highest mean contribution in BLMGTB, whereas 2013Ark19i was concentrated primarily in BLEGS. *Pseudomonas* and *Agrobacterium* were represented more strongly in gill groups, while HIMB11, *Candidatus Aquiluna*, and *Paracoccus* showed their highest mean contributions in BLEGTB. *Phaeodactylibacter* was concentrated primarily in BLMGTB. To provide greater transparency regarding within-group variability, the complete genus-level differential-test output, including group means, standard deviations, nominal *p* values, and adjusted *p* values, is provided in Supplementary Table S3, while the individual pooled-sample relative abundances of the 10 genera displayed in Figure 6 are provided in Supplementary Table S4.

Similarly, 10 ASVs showed nominal group-level *p* values below 0.05 (Figure S5). After Benjamini–Hochberg FDR correction across the complete ASV-level test set, these ASVs were interpreted according to their adjusted q values and were retained as exploratory candidates when FDR significance was not achieved. ASV5 (*p* = 0.03330), ASV17 (*p* = 0.03209), ASV572 (*p* = 0.03432), ASV4 (*p* = 0.03965), ASV6 (*p* = 0.01075), ASV180 (*p* = 0.04195), ASV81 (*p* = 0.02871), ASV80 (*p* = 0.01677), ASV172 (*p* = 0.04902), and ASV79 (*p* = 0.01920). ASV80 showed its strongest representation in BLEGS, ASV79 and ASV180 in BLMGTB, and ASV172 in BLLGTB. Because these comparisons used unadjusted *p* values, they are treated as exploratory group-associated features rather than pairwise-confirmed biomarkers. ASV-level examination further showed that the strong *Limimaricola* signal in middle-stage skin mucus overlapped directly with the supplied-seawater reference. ASV79, assigned to *Limimaricola*, represented 70.89% of the bacterial community in BLY_ST1 and was also the dominant *Limimaricola* ASV in the three Mucus-Mid pools, contributing 71.11%, 24.59%, and 47.14% in BLMGTB1, BLMGTB2, and BLMGTB3, respectively. In contrast, ASV79 represented only 0.20% of BLY_ST2. Thus, the same ASV occurred at high relative abundance in one supplied-seawater sample and in middle-stage skin mucus, indicating that variation in microbial input from supplied seawater is a plausible contributor to the observed Mucus-Mid composition. Because the supplied-seawater samples originated from independent delivery events and could not be temporally matched to the fish sampling stages, these observations do not establish direct source–sink transmission.

### 3.6. Gill Microbiota Shifted from Intermittent-Dominated Toward Persistent-Enriched Abundance

**Figure 7:**
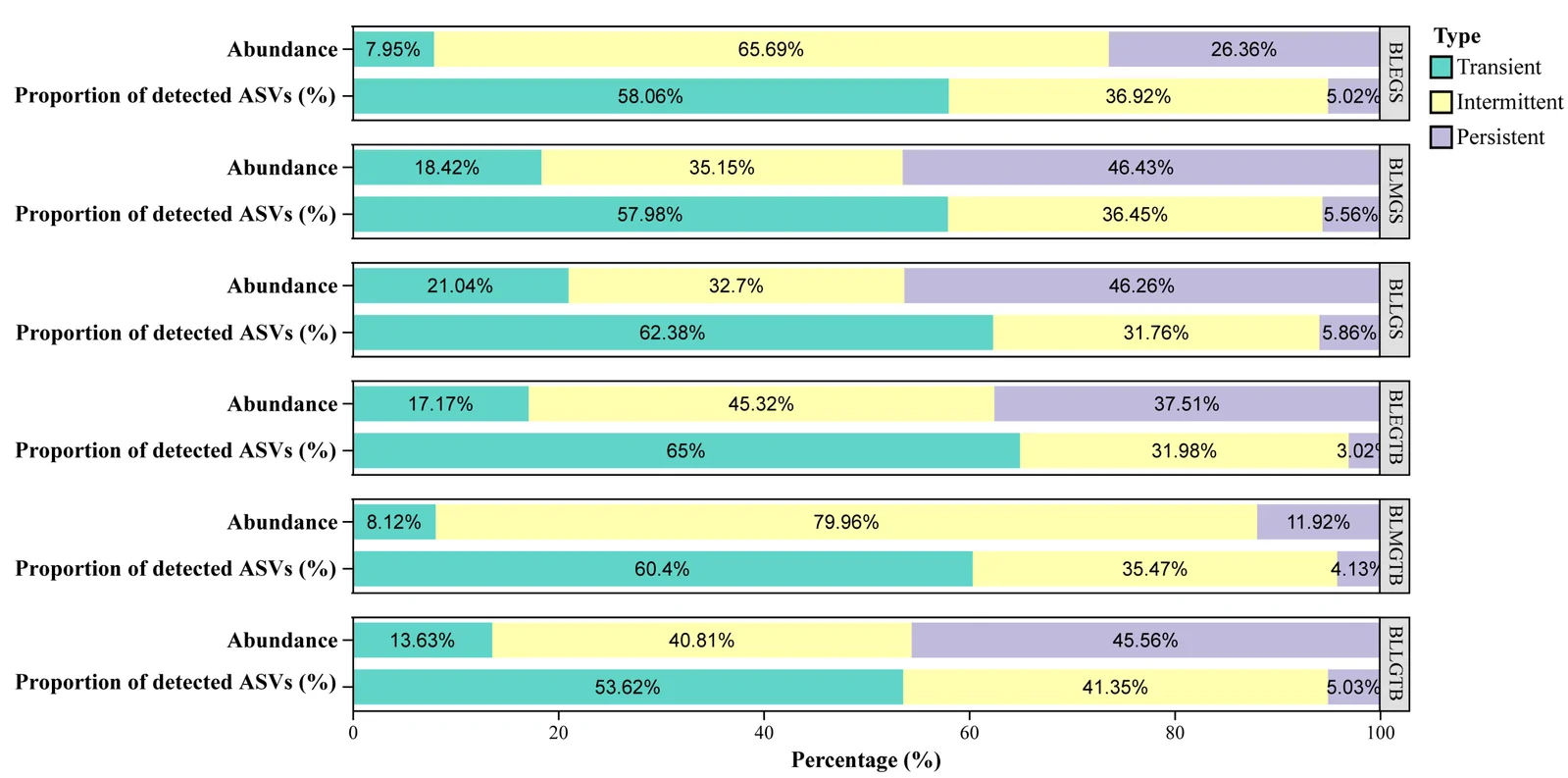
Relative numerical representation and abundance contribution of transient, intermittent, and persistent ASVs across six host-associated groups of D. maruadsi. ASV prevalence was calculated across the 18 host-associated samples, excluding the two supplied-seawater reference samples. ASVs were classified as transient (prevalence < 0.2), intermittent (0.2 ≤ prevalence ≤ 0.8), or persistent (prevalence > 0.8). For each host-associated group, “Number” indicates the percentage of detected ASVs assigned to each occurrence category, whereas “Abundance” indicates the cumulative relative abundance contributed by ASVs in that category. Values shown in Figure 7 represent group-level summaries. The corresponding occurrence-frequency proportions and relative-abundance contributions for each individual pooled sample are provided in Supplementary Table S6.

Dataset-wide occurrence classification revealed a pronounced mismatch between ASV number and abundance contribution in the gill (Figure 7). In BLEGS, transient ASVs represented 58.06% of detected ASVs, intermittent ASVs 36.92%, and persistent ASVs only 5.02%. Despite their small numerical fraction, persistent ASVs contributed 26.36% of total abundance. Intermittent ASVs dominated abundance at 65.69%, whereas transient ASVs contributed only 7.95%.

The number-based composition changed little in BLMGS: transient, intermittent, and persistent ASVs represented 57.98%, 36.45%, and 5.56% of ASV number, respectively. Their abundance contributions, however, changed markedly. Persistent ASVs increased to 46.43% of total abundance and became the largest abundance component, while intermittent ASVs declined to 35.15% and transient ASVs increased to 18.42%. Thus, the early-to-middle transition involved redistribution of abundance among occurrence classes rather than a major change in the numerical size of those classes.

BLLGS retained a similar occurrence structure. Transient, intermittent, and persistent ASVs represented 62.38%, 31.76%, and 5.86% of ASV number, respectively. Persistent ASVs nevertheless contributed 46.26% of abundance, whereas intermittent and transient ASVs contributed 32.70% and 21.04%. Across all three gill stages, the persistent fraction therefore remained numerically small, but its abundance contribution increased by approximately 20 percentage points from BLEGS to BLMGS and remained at a similar level in BLLGS. The principal gill pattern was consequently an early-to-middle redistribution from intermittent-dominated abundance toward greater quantitative contribution from a small, widely occurring ASV fraction.

To evaluate whether the pronounced middle-stage skin-mucus pattern was driven by a single pooled sample, occurrence-class abundance contributions were additionally examined at the individual-pool level. ASVs classified as intermediate-prevalence members across the combined host-associated dataset contributed 86.27%, 76.50%, and 77.03% of the total relative abundance in BLMGTB1, BLMGTB2, and BLMGTB3, respectively. Thus, the strong intermediate-prevalence contribution was consistently observed across all three middle-stage skin-mucus pools rather than being attributable solely to BLMGTB1, although substantial taxonomic differences remained among individual pools. Tissue-specific prevalence analyses produced different quantitative occurrence–abundance profiles from the combined host-wide analysis. When prevalence was defined within the nine gill samples, persistent ASVs contributed 67.02%, 48.16%, and 50.41% of abundance in the early, middle, and late gill stages, respectively. When prevalence was defined within the nine skin-mucus samples, persistent ASVs contributed 50.80%, 78.05%, and 54.44%, respectively. Thus, occurrence-category interpretation depended substantially on the sample set used to define prevalence. In contrast, repeating the combined 18-host analysis using alternative 0.25/0.75 prevalence thresholds retained the same broad host-wide direction: persistent-ASV abundance increased from 29.99% to 49.72% and 49.08% across gill stages, whereas skin-mucus values changed from 42.30% to 24.90% and then 47.59%.

### 3.7. Skin Mucus Displayed a Non-Linear Occurrence–Abundance Pattern with a Distinct Middle Stage

Skin mucus followed a different occurrence–abundance pattern (Figure 7). In BLEGTB, transient ASVs represented 65.00% of ASV number, intermittent ASVs 31.98%, and persistent ASVs 3.02%. Persistent ASVs nevertheless contributed 37.51% of total abundance, while intermittent and transient ASVs contributed 45.32% and 17.17%, respectively.

BLMGTB showed the most pronounced redistribution among the three skin-mucus stages. Transient, intermittent, and persistent ASVs accounted for 60.40%, 35.47%, and 4.13% of ASV number, respectively, values that differed only modestly from BLEGTB. In contrast, abundance contributions changed sharply. Intermittent ASVs accounted for 79.96% of total abundance, whereas persistent ASVs declined to 11.92% and transient ASVs to 8.12%. The middle-stage shift therefore reflected a major change in abundance dominance rather than a large expansion in the number of intermittent ASVs.

BLLGTB showed a second redistribution. Transient, intermittent, and persistent ASVs represented 53.62%, 41.35%, and 5.03% of ASV number, respectively. Persistent ASVs again became a major abundance component at 45.56%, while intermittent and transient ASVs contributed 40.81% and 13.63%. Thus, skin mucus showed a stage-specific redistribution of occurrence classes: persistent-ASV abundance decreased markedly from the early to the middle stage and subsequently increased at the late stage, whereas intermittent-ASV abundance showed the opposite pattern.

Direct comparison of the two mucosal habitats highlighted their contrasting paths. From the early to middle stage, persistent-ASV abundance increased from 26.36% to 46.43% in the gill but decreased from 37.51% to 11.92% in skin mucus. Over the same transition, intermittent abundance declined from 65.69% to 35.15% in the gill but increased from 45.32% to 79.96% in skin mucus. By the late stage, persistent ASVs contributed similar fractions of total abundance in BLLGS (46.26%) and BLLGTB (45.56%), but the paths leading to these similar endpoints were substantially different.

## 4. Discussion

### 4.1. Gill and Skin-Mucus Microbiota Followed Distinct Growth-Associated Trajectories

The central finding of this study is that the gill and skin-mucus microbiota of cultured *D. maruadsi* were both reorganized across growth stages, but the structure of that reorganization differed between the two mucosal habitats. Significant overall differentiation in PCoA, extensive ASV-level turnover, contrasting genus-level patterns, and divergent occurrence–abundance structures all indicate that the early-, middle-, and late-stage communities were not interchangeable. Similar temporal or age-associated restructuring has been reported in the external mucosal microbiota of Pacific chub mackerel (*Scomber japonicus*) [15], European seabass (*Dicentrarchus labrax*) and gilthead seabream (*Sparus aurata*) [16], yellowtail kingfish (*Seriola lalandi*) [17], and Atlantic salmon (*Salmo salar*) [18]. Together, these observations support the view that external mucosal microbiota remain dynamic during fish growth.

The present design reduces several sources of between-group variation because the same cohort was continuously reared in the same culture pond and received an unchanged feed formulation. Nevertheless, growth stage covaried with cultivation duration, host physiological development, feeding quantity, and cumulative environmental exposure. Temporal and developmental variation in external mucosal microbiota has been reported across several marine fishes, although the specific patterns differ among systems. Minich [15] identified temporal, environmental, and biological drivers of the mucosal microbiome in wild *Scomber japonicus*. Rosado reported age-associated changes in the skin and gill microbiota of farmed seabass and seabream [16]. In yellowtail kingfish, Minich showed that water, feed, and the hatchery environment contributed to mucosal microbiome colonization across ontogeny [17]. Lorgen-Ritchie further documented temporal changes in Atlantic salmon skin and gill microbiota under recirculating aquaculture conditions [18]. Together, these studies support a dynamic view of external mucosal microbiota while also emphasizing the simultaneous contributions of host development and rearing environment [1,2,3].

The divergence between gill and skin-mucus patterns is biologically plausible because the two surfaces provide different microhabitats. The gill performs gas exchange, osmoregulation, ion transport, acid–base regulation, and nitrogenous waste excretion [5], whereas skin mucus forms a continuously renewed barrier exposed directly to the aquatic environment [3,9,10]. Previous studies have similarly shown that gill and skin microbial communities can differ within the same host or culture system [8,11,17,18]. The present results extend this niche-specific perspective by showing that the two habitats also differed in how microbial occurrence classes were redistributed across host growth.

### 4.2. High-Level Taxonomic Conservation Masked Extensive Fine-Scale Turnover

*Pseudomonadota* remained the dominant phylum across most samples, and *Alphaproteobacteria* and *Gammaproteobacteria* constituted major class-level components. Similar dominance of proteobacterial lineages is common in fish skin and gill microbiota [4,7,11,15]. At coarse taxonomic resolution, this pattern could suggest substantial continuity across growth stages. The finer-scale analyses demonstrate otherwise. Nineteen phyla, 57 families, and 85 genera were shared across all seven groups, but only 32 ASVs were universally shared, and each group contained hundreds of group-specific ASVs.

This resolution-dependent pattern is consistent with comparative fish microbiome studies showing that host species, anatomical site, habitat, and environmental exposure can generate stronger differentiation at lower taxonomic resolution than is visible at the phylum level [6,8,11,12,13]. Members of the same broad bacterial lineage can differ in colonization ability, environmental tolerance, substrate use, and host association. Consequently, persistence of a *Pseudomonadota*-dominated framework does not imply stability of the underlying microbial populations.

The particularly large group-specific ASV pool in BLEGTB further illustrates this distinction. Early stage skin mucus contained 932 unique ASVs, approximately twice the number observed in each gill group. Group-specific ASVs are defined by detection patterns and do not, by themselves, establish ecological specialization. Their large number instead indicates that early-stage skin mucus contained a broad fine-scale component that was not consistently represented in the other groups. The heatmap and occurrence-frequency analysis support the same general conclusion: much of the community restructuring occurred through redistribution of individual ASVs within a broadly conserved higher-level taxonomic framework.

### 4.3. Gill Reorganization Was Characterized by Increasing Abundance Contribution from High-Prevalence ASVs

Occurrence-frequency analysis revealed one of the clearest directional patterns in the gill. Persistent ASVs represented only 5.02–5.86% of detected ASVs across all three stages, yet their abundance contribution increased from 26.36% in BLEGS to 46.43% in BLMGS and remained similarly high at 46.26% in BLLGS. Because the numerical size of the persistent fraction changed little, the shift primarily reflected increasing abundance of a relatively small set of widely occurring ASVs rather than recruitment of many additional persistent members.

The physiological characteristics of the gill provide an ecological context for this pattern. Gill surfaces remain in continuous contact with water but differ strongly from the bulk water environment because of active gas exchange, ion transport, acid–base regulation, osmoregulatory processes, and nitrogenous waste excretion [5]. Reef-fish gills can support distinct microbiota despite continual seawater exposure [4], and comparative studies show that gill communities can retain environmental signatures without simply reproducing the surrounding water microbiota [6,7]. The present pattern is therefore consistent with growth-associated changes in local gill conditions altering the quantitative importance of bacterial populations that are broadly distributed across the dataset.

Increasing persistent-ASV abundance does not, however, demonstrate increasing temporal stability. Prevalence here measures recurrence across the 18 host-associated samples rather than residence of the same microbial population within repeatedly sampled individual fish. Transient ASVs also remained numerically dominant and increased in abundance from 7.95% to 21.04% across the gill stages. The later-stage gill community therefore combined a large low-prevalence pool with a comparatively small but quantitatively important high-prevalence component.

The sensitivity analysis using alternative prevalence thresholds yielded the same broad host-wide abundance-redistribution pattern. Using cut-offs of <0.25, 0.25–0.75, and >0.75, persistent-ASV abundance in the gill changed from 29.99% in the early stage to 49.72% in the middle stage and 49.08% in the late stage. In skin mucus, the corresponding values were 42.30%, 24.90%, and 47.59%, respectively. Thus, although the absolute contribution assigned to each occurrence category varied with threshold choice, the principal stage-associated host-wide pattern was not dependent on the exact 0.2/0.8 cutoffs.

### 4.4. Skin Mucus Underwent Pronounced Intermediate-Stage Restructuring

Skin mucus showed a marked stage-specific redistribution of occurrence classes. Persistent-ASV abundance decreased from the early to the middle stage and subsequently increased at the late stage, whereas intermediate-prevalence ASVs showed the opposite temporal pattern. The middle-stage skin-mucus community therefore represented a distinct occurrence–abundance configuration rather than a simple midpoint between the early and late groups.

Independent results support this interpretation. BLMGTB occupied a distinct region of PCoA space and showed pronounced representation of *Limimaricola*, while ASV79 and ASV180 also reached their highest mean abundance in this group. Agreement among ordination, genus-level composition, ASV-level patterns, and occurrence-frequency structure makes the middle-stage shift one of the most internally consistent features of the dataset. Fish skin mucus is a continuously renewed microbial habitat and can respond rapidly to changes in host condition and environmental exposure [3,9]. Previous work has documented substantial skin-microbiome changes associated with salinity transition, culture conditions, and temporal variation [9,10,15,18]. These observations provide biological context for the strong restructuring observed in BLMGTB without establishing a specific causal mechanism. The strong *Limimaricola* signal in middle-stage skin mucus requires particular caution because the same dominant ASV (ASV79) was also highly abundant in one supplied-seawater reference. Skin mucus is continuously exposed to surrounding water, making environmental immigration a plausible contributor to its microbial composition. However, the supplied-seawater samples originated from independent tanker-delivery events and were not temporally matched to the three fish sampling stages, and pond water itself was not sampled. Consequently, the relative contributions of environmental microbial input, host-associated selection, and other temporal changes cannot be separated. The middle-stage mucus configuration should therefore be regarded as an observed community state rather than evidence of a host-driven developmental transition.

BLLGTB was not simply a return to the early-stage skin-mucus state. Although persistent ASVs contributed similar abundance fractions in BLEGTB and BLLGTB, their taxonomic structures differed. BLEGTB contained the largest host-associated group-specific ASV pool, whereas BLLGTB2 and BLLGTB3 showed strong *Photobacterium* representation and ASV172 was most abundant in BLLGTB. The late-stage community is therefore better described as a reassembled configuration in which the persistent fraction again became quantitatively important. *Photobacterium* showed marked enrichment in two late-stage skin-mucus pools, accounting for approximately 84% and 63% of the communities in BLLGTB2 and BLLGTB3, respectively, whereas its contribution was minimal in BLLGTB1. No abnormal mortality, overt clinical signs, or disease-related treatments were recorded at the late-stage sampling. Moreover, 16S rRNA gene amplicon sequencing does not resolve pathogenic potential at the strain level. The high relative abundance of *Photobacterium* is therefore interpreted as a compositional feature of these late-stage mucus communities rather than as evidence of infection or disease.

### 4.5. Occurrence–Abundance Decoupling Revealed Community Organization Beyond Diversity Metrics

A consistent feature of both mucosal habitats was the mismatch between the numerical size of occurrence classes and their abundance contribution. Persistent ASVs generally represented only approximately 3–6% of detected ASVs but contributed up to approximately 46% of total abundance, whereas transient ASVs frequently represented more than half of detected ASVs while contributing a much smaller fraction of abundance. Similar abundance–occupancy relationships occur in microbial communities in which widely distributed taxa are few in number but disproportionately abundant [19].

This distinction adds information that conventional diversity and ordination analyses do not provide. PCoA establishes that communities differ among groups, but it does not identify whether those differences are associated mainly with high-, intermediate-, or low-prevalence members. In the gill, the principal abundance redistribution involved increasing quantitative importance of high-prevalence ASVs. In skin mucus, the most pronounced transition involved temporary dominance of intermediate-prevalence ASVs in the middle stage. These contrasting occurrence–abundance patterns therefore represent an ecological dimension of community reorganization that is complementary to taxonomic turnover.

A persistent ASV was defined by occurrence in more than 80% of the 18 host-associated samples, corresponding to detection in at least 15 samples. Under this operational definition, persistent ASVs represent dataset-wide recurrent populations rather than experimentally demonstrated permanent colonists. The prevalence thresholds used here should therefore be regarded as operational analytical choices rather than intrinsic ecological boundaries. The alternative-threshold analysis showed that changing the cut-offs affected the absolute proportions assigned to each occurrence category but did not eliminate the principal host-wide abundance-redistribution pattern.

### 4.6. Group-Associated Genera and ASVs Provide Taxonomic Anchors Without Establishing Function

Several candidate genera help anchor the broader community changes taxonomically. 2013Ark19i was strongly represented in BLEGS, whereas *Limimaricola* reached its highest mean abundance in BLMGTB. *Phaeodactylibacter*, HIMB11, *Candidatus Aquiluna*, *Nautella*, *Pseudomonas*, *Agrobacterium*, *Winogradskyella*, and *Paracoccus* showed additional group-associated patterns. The ASV-level results provided even more localized signals, with ASV80 associated with BLEGS, ASV79 and ASV180 with BLMGTB, and ASV172 with BLLGTB.

These features represent taxonomic indicators of community reorganization rather than direct evidence of microbial function. Amplicon sequencing based on the 16S rRNA gene describes taxonomic composition and relative abundance but does not directly resolve microbial activity, strain-level physiology, or causal effects on the host [1,2,3]. This limitation is particularly relevant for broad genera containing ecologically diverse members.

The taxon-level comparisons should be interpreted cautiously, accordingly, the genera and ASVs highlighted here represent exploratory candidates that provide taxonomic context for the broader community patterns rather than independently validated differential taxa. Their stage- or tissue-associated distributions require confirmation in datasets with greater biological replication and multiple-testing-adjusted statistical frameworks.

### 4.7. Study Limitations and Integrated Interpretation

The study design imposes several boundaries on interpretation. First, the three groups represent successive samples from the same cohort but not repeated sampling of the same individual fish. Growth stage therefore covaries with cultivation duration and developmental status. Second, each host-associated sample was a pool derived from five fish. The statistical unit is the pooled sample, and the present data do not resolve inter-individual variation among fish within each pool. Third, only two supplied-seawater samples were available, limiting inference about environmental source dynamics. Forth, although DNA extraction blanks and PCR negative controls were included during laboratory processing, these controls were not sequenced. Potential low-level reagent-associated contamination therefore cannot be completely excluded, particularly for relatively low-biomass mucosal samples. Taxa that may also occur in laboratory reagent backgrounds are consequently interpreted cautiously, and their detection alone is not considered evidence of specific host association. Fifth, supplied seawater should be regarded as a potential microbial source rather than solely as an external reference. Marine surfaces are rapidly colonized by microorganisms from surrounding seawater, and environmental immigration can therefore contribute to surface-associated community assembly. This possibility is particularly relevant to the high abundance of *Limimaricola* ASV79 observed in both BLY_ST1 and middle-stage skin mucus. Nevertheless, because contemporaneous tank-water samples were unavailable, direct source tracking cannot be performed in the present study. Finally, 16S rRNA gene sequencing does not directly measure gene expression, metabolic activity, or functional effects on the host.

Within these limits, multiple analyses converged on a coherent tissue-specific pattern. Gill reorganization was characterized mainly by an early-to-middle increase in abundance contribution from dataset-wide persistent ASVs, followed by maintenance of a similar persistent contribution at the late stage. Skin mucus followed a non-linear sequence, with a pronounced intermittent-dominated middle-stage state and subsequent late-stage reassembly. Both habitats retained broad high-level taxonomic similarity while undergoing extensive genus- and ASV-level turnover. The external mucosal microbiome of *D. maruadsi* is therefore better described as two distinct host-associated microbial habitats with different growth-associated occurrence–abundance dynamics rather than as a single surface microbiome undergoing uniform maturation.

## 5. Conclusions

Gill and skin-mucus microbiota of cultured *D. maruadsi* showed substantial compositional restructuring across three successive growth-associated sampling stages. Factorial multivariate analysis identified significant effects of mucosal tissue, sampling stage, and their interaction, supporting different stage-associated community responses in the two external mucosal compartments. Broad taxonomic composition remained largely *Pseudomonadota*-dominated, whereas extensive turnover was evident at finer taxonomic resolutions. Skin mucus also showed a significant decline in richness across successive stages, whereas no corresponding alpha-diversity change was detected in the gill. Host-wide prevalence analysis further showed that the two tissues differed in how they weighted ASVs that were broadly recurrent across the combined host-associated dataset. However, tissue-specific sensitivity analyses demonstrated that the quantitative contribution of prevalence categories depended on the sample set used for classification. These occurrence categories should therefore be interpreted as operational descriptors rather than intrinsic measures of temporal persistence within each tissue. The strong overlap of a dominant *Limimaricola* ASV between one supplied-seawater sample and middle-stage skin mucus further indicates that environmental microbial input may contribute to some observed compositional patterns. Because the study followed a single cohort in one culture system and growth stage covaried with cultivation time and unmeasured environmental variation, the observed differences cannot be attributed causally to host growth alone. The present study therefore provides a descriptive baseline for stage-associated variation in the external mucosal microbiota of cultured *D. maruadsi* and identifies tissue-dependent patterns that warrant validation using replicated culture units, contemporaneous environmental sampling, and greater biological replication.

## Data Availability

The raw 16S rRNA gene amplicon sequencing data generated in this study are openly available in the Genome Sequence Archive (GSA), National Genomics Data Center, China National Center for Bioinformation/Beijing Institute of Genomics, Chinese Academy of Sciences, under accession number CRA049855.

## Supplementary Materials

The following supporting information can be downloaded at: https://www.mdpi.com/article/10.3390/vetsci13101073/s1, Figure S1: Rarefaction curves for (A) Chao richness, (B) Shannon diversity, and (C) Good’s coverage across gill, skin-mucus, and supplied-seawater samples of *D. maruadsi*; Figure S2: Class-level microbial community composition across gill, skin-mucus, and supplied-seawater samples of *D. maruadsi*; Figure S3: Order-level microbial community composition across gill, skin-mucus, and supplied-seawater samples of *D. maruadsi*; Figure S4: Family-level microbial community composition across gill, skin-mucus, and supplied-seawater samples of *D. maruadsi*; Figure S5: Candidate ASVs showing nominal group-level differences among gill and skin-mucus groups of *D. maruadsi*. Table S1: Sequence-processing statistics for the 20 gill, skin-mucus, and supplied-seawater samples of *D. maruadsi*; Table S2: Stage-specific and shared ASV patterns within the gill and skin-mucus microbiota of *D. maruadsi*; Table S3: Complete genus-level differential-test output among the six host-associated groups of *D. maruadsi*; Table S4: Sample-level relative abundances of the 10 genera displayed in Figure 6; Table S5: Host-wide persistent ASVs identified across the 18 host-associated samples of *D. maruadsi*; Table S6: Sample-level occurrence-frequency composition and relative-abundance contributions of transient, intermittent, and persistent ASVs across the 18 host-associated samples.
