# Supplementary material for: Growth-Associated Reorganization of Gill and Skin-Mucus Microbiota in Cultured Decapterus maruadsi: Contrasting Occurrence–Abundance Patterns
Source: Vet Sci. 2026 Oct 10;13(10):1073. doi: 10.3390/vetsci13101073 (PMC13709428; doi:10.3390/vetsci13101073)
Supplement: Supplementary file 1 [file vetsci-13-01073-s001.zip › Supplementary Figures/Figure S1. Rarefaction curves for (A) Chao richness, (B) Shannon diversity, and (C) Good’s coverage across gill, skin-mucus, and supplied-seawater samples of D. maruadsi..pdf]

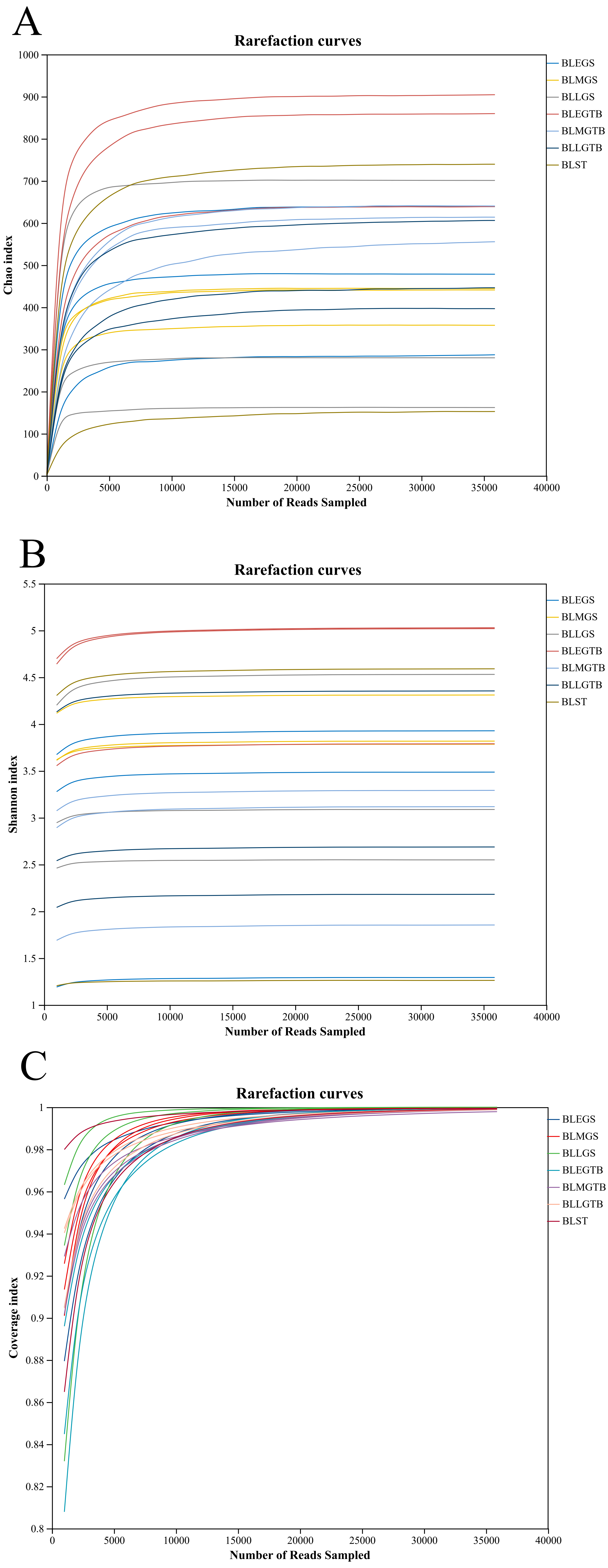

A
Rarefaction curves
Chao index
Number of Reads Sampled
0
100
200
300
400
500
600
700
800
900
1000
5000
10000
15000
20000
25000
30000
35000
40000
BLEGS
BLMGS
BLLGS
BLEGTB
BLMGTB
BLLGTB
BLST
B
Rarefaction curves
Shannon index
Number of Reads Sampled
1
1.5
2
2.5
3
3.5
4
4.5
5
5.5
0
5000
10000
15000
20000
25000
30000
35000
40000
BLEGS
BLMGS
BLLGS
BLEGTB
BLMGTB
BLLGTB
BLST
C
Rarefaction curves
Coverage index
Number of Reads Sampled
0.8
0.82
0.84
0.86
0.88
0.9
0.92
0.94
0.96
0.98
1
0
5000
10000
15000
20000
25000
30000
35000
40000
BLEGS
BLMGS
BLLGS
BLEGTB
BLMGTB
BLLGTB
BLST
