# Supplementary material for: Growth-Associated Reorganization of Gill and Skin-Mucus Microbiota in Cultured Decapterus maruadsi: Contrasting Occurrence–Abundance Patterns
Source: Vet Sci. 2026 Oct 10;13(10):1073. doi: 10.3390/vetsci13101073 (PMC13709428; doi:10.3390/vetsci13101073)
Supplement: Supplementary file 1 [file vetsci-13-01073-s001.zip › Supplementary Figures/Figure S2. Class-level microbial community composition across gill, skin-mucus, and supplied-seawater samples of D. maruadsi..pdf]

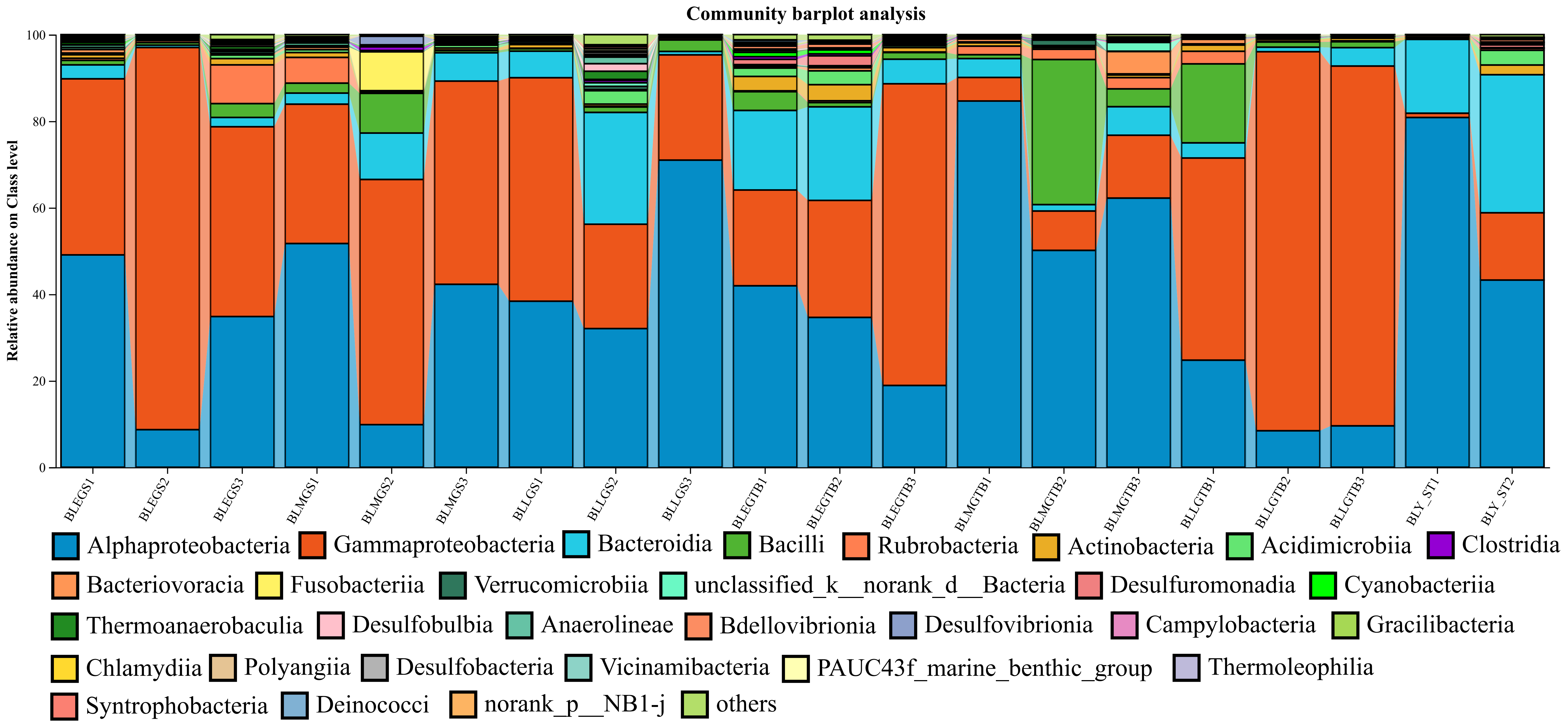
Community barplot analysis
Relative abundance on Class level
0
20
40
60
80
100
BLEGS1
BLEGS2
BLEGS3
BLMGS1
BLMGS2
BLMGS3
BLLGS1
BLLGS2
BLLGS3
BLEGTB1
BLEGTB2
BLEGTB3
BLMGTB1
BLMGTB2
BLMGTB3
BLLGTB1
BLLGTB2
BLLGTB3
BLY_ST1
BLY_ST2
Alphaproteobacteria
Gammaproteobacteria
Bacteroidia
Bacilli
Rubrobacteria
Actinobacteria
Acidimicrobiia
Clostridia
Bacteriovoracia
Fusobacteriia
Verrucomicrobiia
unclassified_k__norank_d__Bacteria
Desulfuromonadia
Cyanobacteriia
Thermoanaerobaculia
Desulfobulbia
Anaerolineae
Bdellovibrionia
Desulfovibrionia
Campylobacteria
Gracilibacteria
Chlamydiia
Polyangiia
Desulfobacteria
Vicinamibacteria
PAUC43f_marine_benthic_group
Thermoleophilia
Syntrophobacteria
Deinococci
norank_p__NB1-j
others
