# Supplementary material for: Growth-Associated Reorganization of Gill and Skin-Mucus Microbiota in Cultured Decapterus maruadsi: Contrasting Occurrence–Abundance Patterns
Source: Vet Sci. 2026 Oct 10;13(10):1073. doi: 10.3390/vetsci13101073 (PMC13709428; doi:10.3390/vetsci13101073)
Supplement: Supplementary file 1 [file vetsci-13-01073-s001.zip › Supplementary Figures/Figure S4. Family-level microbial community composition across gill, skin-mucus, and supplied-seawater samples of D. maruadsi..pdf]

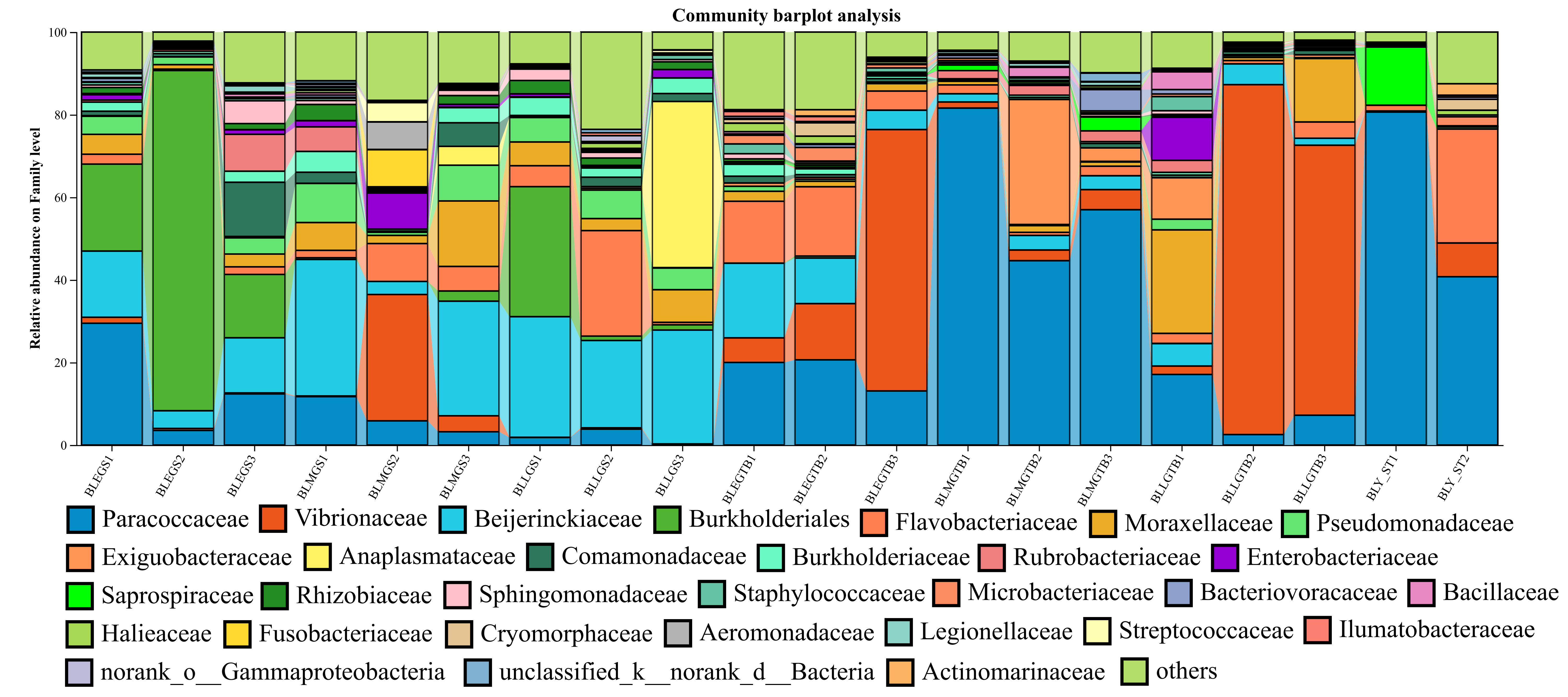

Community barplot analysis
Relative abundance on Family level
0
20
40
60
80
100
BLEGS1
BLEGS2
BLEGS3
BLMGS1
BLMGS2
BLMGS3
BLLGS1
BLLGS2
BLLGS3
BLEGTB1
BLEGTB2
BLEGTB3
BLMGTB1
BLMGTB2
BLMGTB3
BLLGTB1
BLLGTB2
BLLGTB3
BLY_ST1
BLY_ST2
Paracoccaceae
Vibrionaceae
Beijerinckiaceae
Burkholderiales
Flavobacteriaceae
Moraxellaceae
Pseudomonadaceae
Exiguobacteraceae
Anaplasmataceae
Comamonadaceae
Burkholderiaceae
Rubrobacteriaceae
Enterobacteriaceae
Saprospiraceae
Rhizobiaceae
Sphingomonadaceae
Staphylococcaceae
Microbacteriaceae
Bacteriovoracaceae
Bacillaceae
Halieaceae
Fusobacteriaceae
Cryomorphaceae
Aeromonadaceae
Legionellaceae
Streptococcaceae
Ilumatobacteraceae
norank_o__Gammaproteobacteria
unclassified_k__norank_d__Bacteria
Actinomarinaceae
others
