# Supplementary material for: Growth-Associated Reorganization of Gill and Skin-Mucus Microbiota in Cultured Decapterus maruadsi: Contrasting Occurrence–Abundance Patterns
Source: Vet Sci. 2026 Oct 10;13(10):1073. doi: 10.3390/vetsci13101073 (PMC13709428; doi:10.3390/vetsci13101073)
Supplement: Supplementary file 1 [file vetsci-13-01073-s001.zip › Supplementary Figures/Figure S5. Candidate ASVs showing nominal group-level differences among gill and skin-mucus groups of D. maruadsi..pdf]

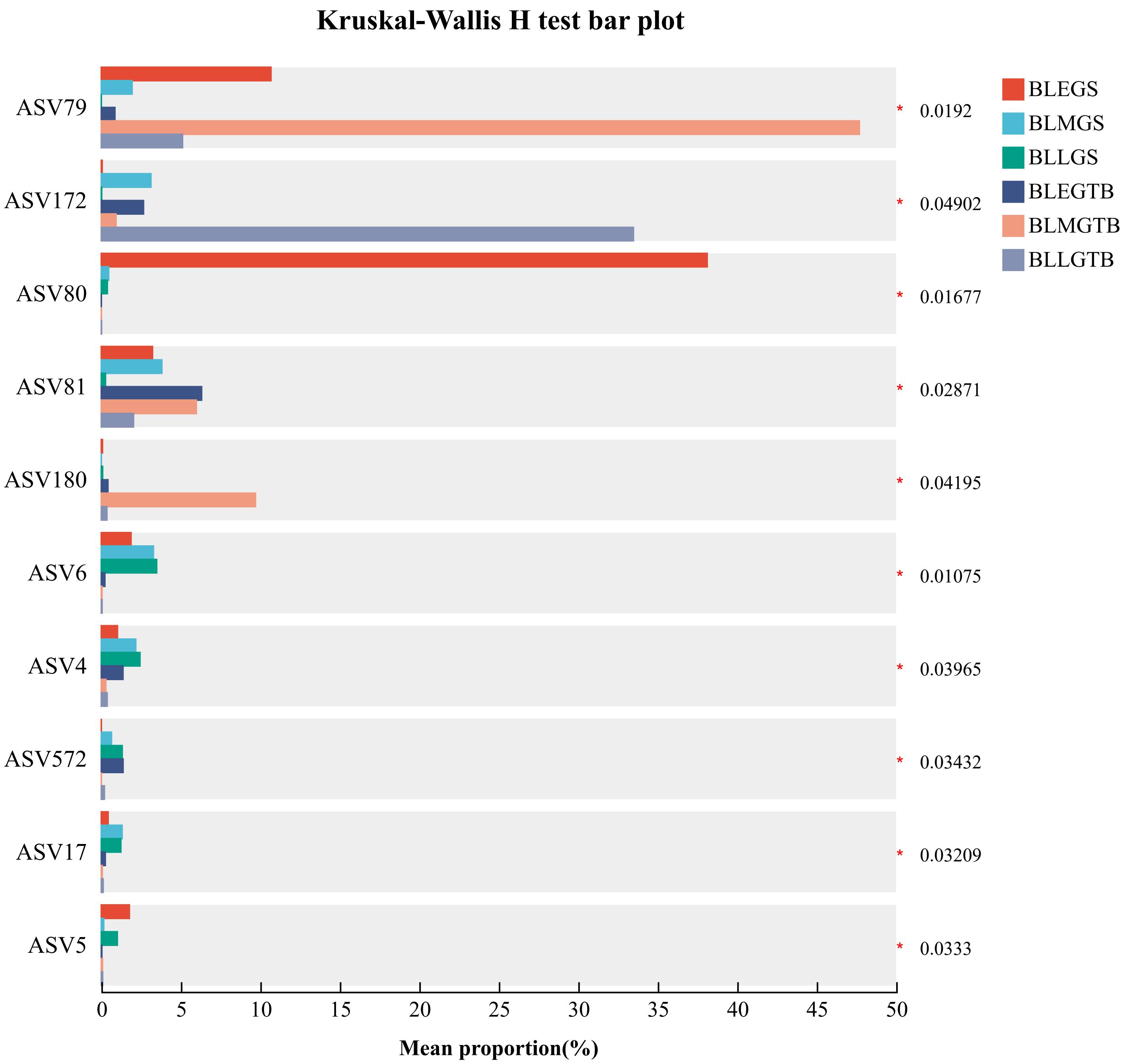

Kruskal-Wallis H test bar plot
ASV79
ASV172
ASV80
ASV81
ASV180
ASV6
ASV4
ASV572
ASV17
ASV5
* 0.0192
* 0.04902
* 0.01677
* 0.02871
* 0.04195
* 0.01075
* 0.03965
* 0.03432
* 0.03209
* 0.0333
BLEGS
BLMGS
BLLGS
BLEGTB
BLMGTB
BLLGTB
0 5 10 15 20 25 30 35 40 45 50
Mean proportion(%)
